# PW01-016 – Are different disease subtypes present in FMF

**DOI:** 10.1186/1546-0096-11-S1-A69

**Published:** 2013-11-08

**Authors:** S Akar, T Kasifoglu, D Solmaz, S Bilge, I Sari, M Tunca

**Affiliations:** 1 Rheumatology, DEUTF, Izmir, Turkey; 2 Rheumatology, Eskisehir Osmangazi University, Eskisehir, Turkey

## Introduction

Familial Mediterranean fever (FMF) is an auto-inflammatory disorder characterized by self limited attacks of fever and serositis. The disease expression may be different in different ethnic groups and patients with certain MEFV mutations may be prone to have more severe disease and a greater probability of developing amyloidosis (1). Recently we showed that amyloidosis is the only predictor of mortality in Turkish FMF patients (2), however clinical subtypes with different clinical and genetic characteristics have been never identified previously.

## Objectives

The aim of this study was to evaluate whether there are clinical subgroups, which may have different prognosis, among FMF patients.

## Methods

The cumulative clinical features of a large group of FMF patients (1168 patients, 575 female [49.2%] and mean age was 35.3 ± 12.4 years) were studied. To analyse our data and identify groups of FMF patients with similar clinical characteristics, a two-step cluster analysis using log-likelihood distance measures was performed. For clustering the FMF patients, we evaluated the following variables: gender, current age, age at symptom onset, age at diagnosis, the presence of major clinical features (fever, peritonitis, pleuritis, arthritis, erysipelas like erythema [ELE], febrile myalgia, amyloidosis), variables related with therapy (the dosage of colchicine, compliance with therapy, and the presence of attacks despite colchicine), the family history for FMF and for renal failure and the presence of M694V allele.

## Results

Three distinct groups of FMF patients were identified. Cluster 1 was characterized by high prevalence of arthritis, pleuritis, ELE, and febrile myalgia. The dosage of colchicine and the frequency of amyloidosis were lower in cluster 1. Patients in cluster 2 had earlier age at symptom onset and diagnosis. Other characteristics of cluster 2 were high frequency of arthritis, amyloidosis, M694V allele and family history for FMF. This group of patients was using highest dose of colchicine. The cluster 3 was characterized by the lowest frequency of M694V allele, ELE, arthritis, protracted febrile myalgia. The colchicine resistance was also lower in cluster 3. The mean age and age at diagnosis was the highest in cluster 3.

## Conclusion

Patients with FMF could be clustered into distinct patterns of clinical and genetic manifestations and these patterns may have different prognostic significance.

## Disclosure of interest

None declared

